# Impact of urban–rural resident basic medical insurance on consumption quality of middle-aged and older adult residents: evidence from rural China

**DOI:** 10.3389/fpubh.2024.1374552

**Published:** 2024-10-03

**Authors:** Yajie Zhou, Junyu Ping

**Affiliations:** ^1^School of Economics, Huazhong University of Science and Technology, Wuhan, China; ^2^School of Optical and Electronic Information, Huazhong University of Science and Technology, Wuhan, China

**Keywords:** urban–rural resident basic medical insurance, time-varying DID model, middle-aged and older adult rural residents, quality of consumption, health economics

## Abstract

In the context of the Chinese government’s advocacy for “Internal Circulation”, ongoing reforms in medical insurance policies raise critical questions about whether the basic medical insurance system can stimulate resident consumption and enhance its quality. Based on five waves of panel data from the China Health and Retirement Longitudinal Survey (CHARLS), this paper analyzes the impact of medical insurance on the consumption scale and structure of middle-aged and older adult rural residents by using the time-varying difference-in-differences (time-varying DID) method. The findings indicate that the Urban–Rural Resident Basic Medical Insurance (URRBMI) effectively stimulates the consumption scale of rural middle-aged and older adult individuals, particularly enhancing non-food consumption, development-oriented spending, and hedonic consumption. This, in turn, optimizes the consumption structure and improves overall consumption quality. Notably, URRBMI significantly enhances the consumption scale and structure among rural middle-aged and older adult women, unmarried individuals, and residents in western China. These results suggest that URRBMI plays a crucial role in alleviating consumption inequality within rural areas and across different regions, providing a theoretical foundation for policy-making.

## 1 Introduction

Chinese government emphasized “focusing on expanding domestic demand and enhancing the fundamental role of consumption in economic development.” In the current context of “Internal Circulation”,^1^ stimulating consumption from various perspectives is essential for fostering economic growth. Contrary to the life cycle theory predictions, a U-shaped relationship exists between the saving rate of Chinese households and the age of household heads (1, 2). Specifically, households with older heads tend to save more, which suppresses their consumption behavior. Based on the life cycle model, Chamon et al. (1) speculated that the turning point of consumption smoothing behavior of Chinese urban and rural residents is around the age of 45, and residents’ precautionary saving motive would gradually weaken after the age of 45. Therefore, the consumption potentiality of middle-aged and older adult residents should be great, which means that they will have greater enthusiasm to spend. Despite the substantial consumption potential of middle-aged and older adult individuals, many households in these people exhibit insufficient consumption and high saving rates. This phenomenon is particularly pronounced among rural residents, who tend to have stronger precautionary saving motives compared to their urban counterparts. The high saving rates observed among many Chinese residents are often attributed to the need for precautionary savings to mitigate future uncertainties (3). Improving the social security system can effectively resist external economic risks, reduce uncertainty and the proportion of precautionary saving, and improve the willingness to consume.

From the perspective of micro-decision-making, basing on the difference in the implementation time of prefecture-level cities, this paper uses the time-varying difference-in-differences (time-varying DID) method. And the data is selected from China Health and Retirement Longitudinal Survey (CHARLS) to study the impact of rural middle-aged and older adult participation in Urban–Rural Resident Basic Medical Insurance (URRBMI) on consumption decision-making. Compared with the implementation time of URRBMI in different cities, it is found that the five weaves of CHARLS data, spanning from 2011 to 2020, encompass the pilot, promotion, and full coverage period of URRBMI.

With the improvement of China’s economic level, consumption upgrading has drawn increasing attention from scholars. Some researchers measure this upgrading through the diversification of consumption (4). However, consumption quality is more frequently assessed by analyzing the proportions of consumption scale and structure, particularly the growth in expenditures on healthcare, culture, entertainment, and hedonic consumption, which are indicative of improved consumption quality (31, 32). This paper adopts this perspective, examining the scale and specific categories of consumption among rural middle-aged and older adult individuals influenced by URRBMI. It emphasizes the trends in the proportions of medical consumption, non-food consumption, development-oriented spending, and hedonic consumption, thereby exploring the overall consumption quality of these people.

A substantial body of existing literature on basic medical insurance primarily focuses on its impact on residents’ health outcomes. Integrating urban and rural health insurance significantly improves the physical and mental wellbeing of rural older adult individuals (5), increasing their trust in medical services (6). Tang explores how the integration of health insurance alleviates health disparities, indicating improvements not only among rural residents but also for urban populations (7). Many studies indicate that basic medical insurance policies can enhance the frequency of medical facility utilization (8–11), can also promote medical advancements (12), increase labor mobility and labor income (8, 13–15). These policies also pay attention to medical insurance costs. There are two main views in the literature on the effect of medical insurance on residents’ consumption (16, 17). Some scholars focus on the impact of medical insurance and economic behavior. Wang hold the idea that health insurance can encourage rural residents to engage in entrepreneurial activities, which shows that insurance can alleviate risks, thereby motivating families to pursue more economic initiatives (18). With the advancement of Chinese basic medical insurance reform, the impact of basic medical insurance on consumption has begun to receive more attention. Some scholars analyzed the data of the New Rural Cooperative Medical System (NCMS) period and believed that it would not reduce the expenditure of residents’ medical expenses (17). Similarly, studies using catastrophe health insurance data have been able to reach similar conclusions. This suggests that such policies have failed to improve the consumption patterns of rural poor households and may even increase consumption inequality among rural households (16, 19). Conversely, other scholars contend that URRBMI can help narrow the disparities in health and wealth, thereby enhancing non-medical consumption, particularly non-food expenditures (14). Taiwan scholars also used the data from National Health Insurance to reach similar conclusions (20). However, this part of the literature does not deeply explore the consumption scale and quality of the less vulnerable groups in rural areas. The improvement of consumption scale and quality of middle-aged and older adult women in rural areas, unmarried middle-aged and older adult people in rural areas, and middle-aged and older adult people in underdeveloped areas in western China can effectively alleviate the consumption inequality within and between rural areas. Moreover, when these articles were published, URRBMI had not yet been implemented nationwide. Many analyses using DID method centered around the 2016 time point. Consequently, these analyses may lack comprehensiveness.

In contrast to the aforementioned literature, this paper offers three key contributions: First, it focuses specifically on the sub-sample of middle-aged and older adult individuals. In China, households with older heads exhibit higher saving rates, which inhibits their consumption behaviors, a phenomenon that contradicts the inverted-U shape predicted by the life cycle theory (1, 2). Theoretically, the consumption potential of these people remains underutilized. Previous studies have predominantly analyzed the broader rural population; this paper seeks to identify pathways for enhancing the consumption scale and quality among rural middle-aged and older adult individuals through the lens of basic medical insurance policy, thereby addressing a gap in the literature. Second, in reference to Goodman-Bacon (21–23), this paper chooses the time-varying DID method to analyze the entire time process of the implementation of the residents’ medical insurance. At the time of the previous literature publication, DID analyses were generally conducted based on the 2016 time point as the URRBMI had not yet achieved nationwide coverage. Many studies relied on propensity score matching (PSM) due to the absence of a treatment group. In contrast, this paper utilizes a time-varying DID model, incorporating five newly released rounds of data from the CHARLS database, resulting in more comprehensive conclusions. Third, this paper provides a more nuanced analysis of the consumption structure influenced by URRBMI among middle-aged and older adult rural residents. While previous literature has discussed consumption quality based on the distinction between medical and non-medical consumption, as well as food and non-food expenditures, this study further categorizes consumption into subsistence, development, and hedonic categories. The growth in the latter two categories is defined as an improvement in consumption structure. In addition, this paper also delves into the changes in consumption quality of vulnerable groups among the middle-aged and older adult people in rural areas.

## 2 Institutional background

The evolution of basic medical insurance for rural residents in China has undergone three significant phases: the inception of the Rural Cooperative Medical System (RCMS) in 1949, the introduction of the New Rural Cooperative Medical System (NCMS) in July 2003, and the establishment of the Urban–Rural Resident Basic Medical Insurance (URRBMI) in 2016. During the same period, Urban Residents’ Basic Medical Insurance (URBMI) was piloted in 2007, covering urban residents who did not participate in Urban Employee Medical Insurance (UEMI), including students, the older adult, and the unemployed. As the basic medical insurance system has developed, the disparity between urban and rural medical insurance frameworks has exacerbated inequalities within the dual structure of the economy. With the ongoing urbanization and industrialization processes, coupled with the increasing migration of individuals from rural to urban areas, there has been a growing demand for a unified medical insurance system. During the NCMS period, migrant workers were restricted to participating in the NCMS only in their hometowns. This limitation, along with issues such as inconvenient payment processes and complex reimbursement procedures in other regions, hindered the operational efficiency of the NCMS. For policymakers, the challenges presented by urban–rural segmentation, managerial separation, and the dispersion and duplication of resources arising from the dual medical insurance systems have become increasingly pronounced. It is imperative to propose a unified medical insurance system for urban and rural residents. In April 2009, Chinese government proposed to explore the establishment of an integrated basic medical insurance management system for urban and rural areas, and also gradually integrate the management resources of basic medical insurance. This concept marked the first articulation of a vision for an integrated urban–rural basic medical insurance framework. Some provinces and cities began to take the initiative, exploring the integration of NCMS and URBMI. In 2016, Chinese government provided specific guidance for the establishment of URRBMI policy, integrating NCMS and URBMI. URRBMI is an integration of the two basic medical insurance systems for urban and rural residents. Subsequently, the remaining provinces and cities began to respond to this policy, and reform to their own basic medical insurance systems for urban and rural areas. The varying implementation timelines of URRBMI across different regions provide a foundational basis for analyzing its effects. By 2019, the unified medical insurance system for urban and rural residents was fully implemented at the national level, marking a significant milestone in the evolution of medical insurance in China.

**Figure 1:**
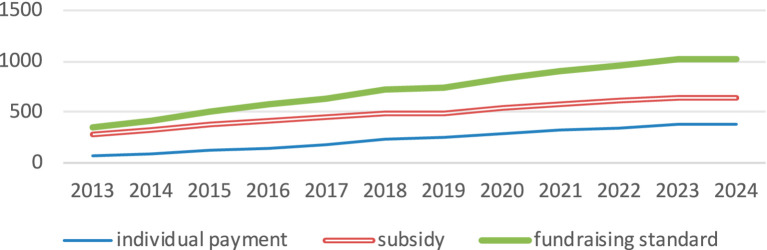
Fundraising standards for rural residents from 2013 to 2024.

The specific guidance for URRBMI put forward the “six unification” of the basic medical insurance integration, which include unified coverage, financing policies, treatment standards, medical insurance catalogs, designated management, and fund management. URRBMI covers all people except those who should be covered by UEMI. The insurance for rural residents was changed from NCMS to URRBMI. It can be seen from Figure 1 that the amount of individual contributions increased after the unification of financing policies, which brought a certain insurance burden to some rural residents. But the financial subsidies for rural residents have also increased significantly, which can offset part of the economic pressure of these residents. From 2013 to 2024, the rate of fundraising has outpaced individual contributions, enabling rural residents to benefit from a higher overall fundraising standard than previously available. In addition, the medical insurance catalog and treatment of local residents’ medical insurance were adjusted in line with the higher standard of URBMI for urban residents. This policy shift significantly improves coverage for rural residents who previously faced lower treatment standards, allowing them to access the same coverage and payment standards as their urban counterparts. Additionally, while the previous NCMS operated on a county-level pooling basis, URRBMI has expanded this to a city-level pooling system. This transition has broadened the scope of medical treatment available to rural residents, reduced the necessity for seeking care in other locations, and increased the local reimbursement rate. The unified designated management and fund management frameworks have reinforced the enforceability and sustainability of URRBMI.

In recent years, the participation rate in medical insurance in China has remained stable at over 95%, with approximately 70% of participants enrolled in URRBMI. This program is characterized by the largest number of insured individuals, the highest coverage rate, and extensive reach, resulting in a significant number of beneficiaries and high operational efficiency. A large proportion of the insured residents of URRBMI are rural residents. Following the integration of the two prior insurance policies, the rural residents covered under NCMS were merged into URRBMI, ensuring that all rural residents are currently insured under this new system. By 2022, rural residents accounted for more than 70% of the total number of residents covered by URRBMI.^2^ The coverage rate for low-income rural residents and those recently lifted out of poverty has consistently exceeded 99%, supported by numerous preferential and subsidy measures instituted by the government to alleviate the insurance burden on these groups.

## 3 Mechanism analysis

Fisher and Friedman’s precautionary saving theory posits that in the face of future uncertainties, households will adjust their consumption and savings behaviors based on fluctuations in income. Specifically, when household income decreases, families typically reduce consumption expenditures and increase precautionary savings. Conversely, an increase in household income generally leads to higher consumption and a reduction in precautionary savings (24). This theory establishes a significant positive correlation between consumption and income. When uncertainties are mitigated through mechanisms such as insurance, expected savings can decrease even if income remains stable, enabling individuals to feel more inclined to increase their consumption. Health is a critical concern for families, particularly for middle-aged and older adult households in rural areas, where it represents a significant source of uncertainty. Adverse health shocks affecting family members—especially primary earners—can lead to substantial declines in household income and consequently squeeze the budget for daily consumption. Additionally, such health issues often result in considerable medical expenses, leading to an immediate rise in medical consumption. Chronic illnesses can further crowd out other consumption needs over the long term, heightening expectations of future risks and prompting households to increase their precautionary savings while reducing overall consumption. In extreme cases, some middle-aged and older adult families in rural areas may regress into poverty due to health-related expenses, further diminishing their future consumption potential. URRBMI policy improves social medical insurance level, reduces the uncertainty of the insured people in the future, and reduces the risk expectation, thereby releasing part of their precautionary savings originally prepared for medical needs and easing the volatility of household consumption (14).

Unlike the inverted U-shaped relationship between household saving rates and the age of the household head predicted by life cycle theory, a U-shaped relationship is observed in China, where saving rates tend to increase for older age groups (1, 2). Consumption levels decrease among older cohorts, particularly in rural areas where lower health awareness and inadequate medical resources exacerbate the likelihood of health issues. Furthermore, concerns related to aging intensify uncertainties about the future, leading to increased precautionary savings. A good social security system can effectively reduce the uncertainty of the future and release more precautionary savings for consumption expenditure. The URRBMI serves as a vital mechanism for nearly all middle-aged and older adult rural residents to mitigate these risks, making its impact on consumption worthy of attention. Thus, we propose the following hypothesis: Hypothesis 1: The Urban–Rural Resident Basic Medical Insurance (URRBMI) effectively stimulates the consumption scale among rural middle-aged and older adult populations.

Once basic food and safety needs are satisfied, individuals tend to pursue higher-level needs, including social connections, esteem, and self-actualization (25), according to Maslow’s Need Hierarchy Theory. Consequently, as individuals transition from lower-level subsistence consumption to more advanced categories such as non-food consumption, developmental consumption, and hedonic consumption, the overall quality of consumption is significantly enhanced. Meanwhile, the URRBMI policy can reduce consumption and welfare inequality among rural residents. Vulnerable groups in rural areas often harbor more pessimistic expectations regarding future risks due to their increased exposure to uncertainties and limited capacity to absorb risks. As a result, these groups may maintain higher levels of precautionary savings, making them more susceptible to the effects of medical insurance policies. Therefore, we propose Hypothesis 2: URRBMI can effectively alleviate consumption inequality both within rural areas and between different regions.

## 4 Data and measurement model

### 4.1 Data sources

To analyze the micro-level impact of URRBMI, this study employs panel data that integrates both temporal and individual-level information across various cities. The implementation timelines of URRBMI were meticulously compiled from four primary sources: first, local normative documents from the Pkulaw database;^3^ second, searching keywords such as “XX city urban and rural residents basic medical insurance implementation measures notice,” and “XX city urban and rural residents began to participate in the insurance payment notice” through various of search engine; third, implementation dates were inferred from notices issued by provincial and certain prefecture-level city departments, as well as documents from other policy implementation agencies, such as tax bureaus; fourth, the relevant news of URRBMI was searched manually through the data that could not be obtained by the above methods. By screening the news reports of relevant years, the implementation time was logically inferred according to the content of the reports. Based on this data collection process, it was determined that 333 prefecture-level regions or cities initiated the URRBMI policy as early as 2007. By 2020, the nationwide rollout of the medical insurance for urban and rural residents had been completed.

The data of individual and household levels used in this paper are derived from the China Health and Retirement Longitudinal Survey^4^ (CHARLS) in 2011, 2013, 2015, 2018, and 2020.

City-level control variables were sourced from the China City Statistical Yearbook, utilizing data from the year of the survey to ensure consistency. These data were collected from statistical yearbooks and bulletins issued by various provinces and cities.

To enhance the generalizability of the results and mitigate the influence of specific samples, individuals residing in the four municipalities directly under the Central Government (Beijing, Shanghai, Tianjin, and Chongqing) were excluded from the analysis. Additionally, the study applies winsorization at the 99th percentile to eliminate outliers, which will be further verified in the stability check section.

### 4.2 Econometric model

Recent official reports indicate that the coverage rate of URRBMI has surpassed 95%. Within a year of the release of the guidelines on the implementation of medical insurance for local residents, almost all eligible residents in the city participated in the insurance. Based on the above situation, the insured and non-insured families can be assigned according to the location of the household registration and the time of the interview. Insured families were designated as the treatment group, whereas non-insured families served as the control group. It is essential that there are no systematic differences in other characteristics between the two groups, ensuring that the decision to be insured is independent of other family attributes.

The effect of the implementation of the medical insurance policy for urban and rural residents on household consumption can be divided into the “time effect” and “policy treatment effect.” The core principle of the DID method is to disentangle the policy effects from time effects between the treatment and control groups, thereby isolating the “net policy effect” attributable to the differences between the two groups. Given that the implementation timeline of URRBMI varies across cities, the evolving impact of the policy necessitates a time-varying DID model. This study employs consumption data from the CHARLS database for the years 2011, 2013, 2015, 2018, and 2020. This “quasi-natural experiment” was carried out with the residents of the city where the policy was implemented as the treatment group and the residents of the area where the policy was not implemented as the control group. Referring to the study of Beck et al. (26), this paper uses time-varying DID to analyze the impact of the residents’ medical insurance on consumption behavior. Based on the parallel trend test, the robustness test is performed by using the counter-fact hypothesis placebo test, and removing the samples of special years and special cities.


(1) $$\begin{array}{l} Consumption_{i,t}=\\ \alpha_{0}+\beta_{1}Integration_{i,t}+\sum \beta_{k}Controls_{i,t}+\gamma_{i}+\lambda_{t}+\varepsilon_{i,t} \end{array}$$

(2) $$Integration_{i,t}=treat_{i}\times post_{i,t}$$

where *Consumption_i,t_* represents the consumption of individuals in year *t*. This paper encompasses total consumption by rural households, including medical and non-medical expenditures, food and non-food consumption, as well as subsistence, developmental, and hedonic consumption. *Integration_i,t_* is the core explanatory variable, indicating whether URRBMI policy has been implemented in city *i* at time *t*. Equation 2 is the interpretation of this variable. *treat_i_* is the dummy variable of the implementation object of the medical insurance policy. *post_i,t_* is the dummy variable of the implementation time of the medical insurance policy. *β*_1_ is the estimated coefficient, indicating the impact of the policy implementation on residents’ consumption; *Controls_i,t_* represents a set of control variables affecting the consumption scale. *γ_i_* captures individual fixed effects. *λ_t_* is time fixed effects. *ε_i,t_* denotes the random disturbance term.

### 4.3 Variable description

#### 4.3.1 Explained variables

*Consumption_i,t_* is the explained variable in this paper. The Consumption expenditure of rural residents is selected as the explained variable, and the logarithm of it is processed. The questionnaire includes multiple inquiries regarding consumption expenditures. Referring to the practice of Aguiar (27), this paper combines the consumption categories according to the use of consumption, and categorizes them into various segments: medical and non-medical consumption, food and non-food consumption, as well as subsistence, developmental, and hedonic consumption.

Drawing on the ideas of Carroll et al. (28), this study defines an upgrading of the consumption structure as an increase in the proportion of medical, non-food, developmental, and hedonic expenditures relative to total consumption.

#### 4.3.2 Core explanatory variables

*Integration_i,t_* serves as the core explanatory variable, indicating whether city *i* initiated the implementation of URRBMI at time *t*. If a city has adopted this medical insurance policy, *treat_i_* is assigned a value of 1; otherwise, it is 0. The value of *post_i,t_* takes a value of 1 for the years in which the resident medical insurance was implemented or thereafter and 0 for the year preceding implementation.

#### 4.3.3 Control variables

To mitigate potential biases arising from omitted variable effects, this study incorporates a comprehensive set of control variables at both individual and social levels, while also accounting for year and individual fixed effects. The individual-level control variables include gender, age, education, marital status, employment status, family size, the dependency ratio of older adult individuals (aged 65 years and above), household income, pension status, health status, and the presence of chronic diseases. *Gender* is coded as 1 for males and 0 for females. *Age* is derived from the respondent’s birth year, calculated by subtracting the birth year from the year of the interview (2018). Since the survey was conducted in July, the actual age of respondents who were born in the second half of the year was calculated by subtracting 1 year from the approximate age just calculated. *Edu* is quantified based on the years of schooling reported in the questionnaire. *Marriage* is defined as married (coded as 1), regardless of cohabitation status, and 0 otherwise. *Income* represents the total household income, which has been logarithmically processed here. *Work_status* indicates whether the family is in the state of work, and the value is the same as above. *Health* denotes whether the individual is in good health. *Chronic* indicates the presence of chronic diseases, with similar coding. *Familysize* reflects the total number of individuals within the household. *Older adult* quantifies the number of older adult people in a family. This variable can measure the burden of supporting a family. The control variables at the social level are mainly selected local GDP *per capita* data.^5^ This index can show the economic development of the region (Table 1).

**Table 1:** Descriptive statistics of variables.

Variables	Sample size	Average	SD	Minimum	Median	Maximum
Consumption	69,797	9.873	1.559	0.000	10.039	15.830
Integration	69,797	0.551	0.497	0.000	1.000	1.000
Treat	69,797	0.987	0.114	0.000	1.000	1.000
Post	69,797	0.551	0.497	0.000	1.000	1.000
Income	69,797	8.255	3.756	0.000	9.575	19.807
Age	69,797	60.598	10.184	40.000	60.000	90.000
Gender	69,797	0.717	0.667	0.000	1.000	2.000
Marriage	69,797	0.863	0.344	0.000	1.000	1.000
Edu	69,797	2.012	2.060	0.000	1.000	11.000
Work_status	69,797	0.572	0.495	0.000	1.000	1.000
Familysize	69,797	2.474	1.695	1.000	2.000	17.000
Health	69,797	0.580	0.493	0.000	1.000	1.000
Chronic	69,797	0.345	0.475	0.000	0.000	1.000
Older adult	69,797	0.029	0.187	0.000	0.000	4.000
Pgdp	69,797	10.685	0.561	8.842	10.674	12.153
Medical	69,797	4.712	3.905	0.000	6.217	13.998
Nonmedical	69,797	9.707	1.608	0.000	9.901	15.830
Food	69,797	8.179	2.875	0.000	8.962	15.801
Nonfood	69,797	9.280	1.646	0.000	9.429	15.390
Live	69,797	8.593	2.345	0.000	9.230	15.801
Develop	69,797	7.156	2.930	0.000	8.003	13.998
Enjoy	69,797	8.586	1.767	0.000	8.767	15.386

## 5 Empirical results and analysis

### 5.1 Correlation analysis and collinearity test

Table 2 shows the test of Pearson correlation coefficient matrix before regression. The results show that there is a significant positive correlation between the core explanatory variable integration and consumption, which is consistent with the expected hypothesis. In addition, the control variables such as income and age are significantly correlated with consumption at the significance level of at least 1%. However, it is important to note that the correlation coefficient matrix solely reflects the relationship between pairs of variables and does not account for the influence of control variables or potential confounding factors (such as time effects and individual effects). Therefore, these results are preliminary and necessitate further regression analysis to elucidate the specific relationships involved. Additionally, the potential for multicollinearity among variables can be initially assessed by examining whether the absolute values of the correlation coefficients between explanatory variables exceed 0.9.

**Table 2:** Correlation coefficient matrix of variables.

	Consumption	Integration	Income	Age	Gender	Marriage	Edu	Work status	Familysize	Health	Chronic	Older adult	pgdp
Consumption	1.000												
Integration	0.150***	1.000											
Income	0.266***	0.207***	1.000										
Age	−0.236***	0.082***	−0.075***	1.000									
Gender	0.109***	0.315***	0.142***	0.053***	1.000								
Marriage	0.216***	−0.023***	0.087***	−0.331***	0.015***	1.000							
Edu	0.230***	0.541***	0.293***	−0.049***	0.269***	0.061***	1.000						
Work status	0.042***	0.084***	0.159***	−0.275***	0.101***	0.162***	0.106***	1.000					
Familysize	0.104***	−0.196***	0.099***	−0.113***	−0.082***	0.039***	−0.098***	0.020***	1.000				
Health	0.120***	0.188***	0.134***	−0.111***	0.104***	0.064***	0.223***	0.144***	−0.043***	1.000			
Chronic	0.034***	0.003	0.224***	0.076***	0.009**	−0.024***	−0.011***	−0.001	−0.079***	−0.128***	1.000		
Older adult	0.023***	−0.030***	0.063***	−0.093***	0.038***	0.010***	−0.013***	0.044***	0.107***	0.007*	0.030***	1.000	
Pgdp	0.160***	0.403***	0.082***	0.034***	0.144***	0.014***	0.291***	−0.047***	−0.146***	0.140***	−0.078***	−0.024***	1.000

***, **, and * indicate significance at the 1%, 5%, and 10% levels, respectively, with standard deviation in parentheses.

To address potential multicollinearity, a variance inflation factor (VIF) test was conducted. Table 3 shows the multicollinearity test results of the model. It is evident from the table that the VIF values for all variables are below 10, indicating that the selected indicators in this study do not exhibit multicollinearity.

**Table 3:** Collinearity test.

	VIF	1/VIF
Integration	1.692	0.591
Edu	1.555	0.643
Age	1.246	0.803
Income	1.241	0.805
Pgdp	1.239	0.807
Gender	1.146	0.873
Work status	1.146	0.873
Marriage	1.138	0.879
Chronic	1.117	0.896
Health	1.115	0.897
Familysize	1.105	0.905
Older adult	1.026	0.975
MEAN VIF	1.23	.

In this paper, Hausman test and *F*-test are mainly used to determine whether the data in this paper are suitable for the fixed-effect model, random-effect model, or mixed-effect model. The test results are shown in Table 4. The Hausman test and *F*-test statistics significantly reject the null hypothesis, thus supporting the adoption of a fixed-effect model.

**Table 4:** Hausman test/*F*-test.

Hausman test			*F*-test		
Chi^2^ statistic	*p*-value	Result	Chi^2^ statistic	*p*-value	Result
1730.57	0.000	reject	1.62	0.000	reject

### 5.2 Results of empirical analysis

#### 5.2.1 Impact on consumption scale and structure

Table 5 reports the benchmark regression results obtained by estimating Equation 1, revealing a significantly positive average effect of the implementation of URRBMI on the consumption of middle-aged and older adult individuals in rural areas. Table 5 shows the influence of URRBMI on consumption scale, and the results in its column (1) illustrate that the implementation of the medical insurance policy, without the inclusion of individual control variables and only accounting for individual and year fixed effects, does not yield statistically significant results; however, a positive impact is still evident. Columns (2), (3), (4), and (5) of Table 5 show that the introduction of various control variables consistently yields positive and statistically significant results at the 1% level. After adding common control variables such as income, age, and gender in Column (1), the results are strongly significant. In Table 5, the coefficient for the income variable remains the highest across the analyses, underscoring the foundational role of income in driving consumption—an increase in income correlates with an increase in consumption. Conversely, the age variable exhibits insignificant results throughout the analysis, suggesting that age may not be a critical determinant of consumption among middle-aged and older adult rural residents. Regarding gender, the findings indicate a positive impact on consumption for males, albeit modest. The inclusion of marital status and education further amplifies the impact coefficient of the medical insurance policy on consumption. Even after controlling for work status, family size, health status, chronic diseases, and the proportion of older adult individuals, the impact coefficient remains statistically significant, although it does decrease. When the urban GDP level is added as a control variable in column (5), despite its insignificance, the effect of the residents’ insurance policy on consumption increases from 0.03 to 0.032.

**Table 5:** Impact on consumption scale.

	(1)	(2)	(3)	(4)	(5)
Variables	Consumption	Consumption	Consumption	Consumption	Consumption
Integration	0.009 (1.20)	0.048*** (3.97)	0.050*** (5.37)	0.030*** (3.87)	0.032*** (3.04)
Income		0.065*** (18.14)	0.067 *** (24.45)	0.059*** (18.59)	0.059*** (18.58)
Age		0.002 (1.37)	0.001 (0.91)	0.000 (0.33)	0.000 (0.33)
Gender		0.019*** (3.39)	0.005*** (5.19)	0.002** (2.13)	0.002** (2.00)
Marriage			0.134*** (13.40)	0.147*** (14.17)	0.148*** (14.54)
Edu			0.062*** (24.41)	0.055*** (24.65)	0.055*** (27.14)
Work_status				0.075*** (12.19)	0.075*** (12.17)
Familysize				0.145*** (22.25)	0.145*** (22.41)
Health				0.036*** (4.94)	0.036*** (4.91)
Chronic				0.042*** (36.13)	0.042*** (35.81)
Older adult				0.051*** (7.21)	0.051***(7.30)
Pgdp					0.027 (0.73)
Constant	10.075*** (1123.83)	9.529*** (154.06)	9.521*** (227.50)	9.277*** (212.86)	9.564*** (23.69)
Observations	69,797	69,797	69,797	69,797	69,797
R-squared	0.0448	0.0670	0.0698	0.0867	0.0867
Number of groups	35,941	35,941	35,941	35,941	35,941
Ind	YES	YES	YES	YES	YES
Year	Yes	YES	YES	YES	YES

***, **, and * indicate significance at the 1, 5, and 10% levels, respectively, with standard deviation in parentheses.

Table 6 illustrates the relationships among food and non-food consumption, subsistence, developmental, and hedonic consumption, all of which are significantly correlated at the 1% level. This finding corroborates the hypothesis that the consumption patterns of middle-aged and older adult rural residents have improved under the influence of the residents’ medical insurance. In particular, food consumption—classified as subsistence consumption—exhibits a significant negative correlation with subsistence consumption, whereas non-food consumption shows a significant positive correlation with developmental and hedonic consumption, aligning with the expected hypothesis. The impact of URRBMI on medical and non-medical consumption is not significantly correlated, which is inconsistent with the conclusion of Chen et al. (14), which posited a significant positive impact on non-medical consumption. According to the results in Table 6, marital status and health status, particularly the presence of chronic diseases, are more influential factors for middle-aged and older adult individuals in rural areas. Those with chronic diseases require more medical treatment and, consequently, higher medical consumption. When family members are threatened by health risks in marital status, the medical intervention behavior and the medical consumption are more. In contrast, non-medical consumption is more significantly influenced by family size; larger families incur greater daily living expenses, leading to increased non-medical consumption. Overall, the findings suggest that the consumption structure among rural middle-aged and older adult populations is being optimized and enhanced as a result of the residents’ medical insurance.

**Table 6:** Impact on consumption structure.

	(1)	(2)	(3)	(4)	(5)	(6)	(7)
Variables	Medical	Non-medical	Food	Non-food	Live	Develop	Enjoy
Integration	0.040 (1.24)	0.007 (0.68)	−0.125*** (−4.15)	0.058*** (8.75)	−0.065*** (−3.41)	0.101*** (3.88)	0.021*** (2.91)
Income	0.067*** (31.16)	0.061*** (20.93)	0.061*** (21.67)	0.060*** (19.76)	0.062*** (24.02)	0.078*** (28.11)	0.061*** (23.78)
Age	−0.009*** (−5.43)	0.003*** (3.84)	0.007** (2.14)	−0.002* (−1.92)	0.008* (1.93)	0.004** (2.07)	−0.002 (−1.15)
Gender	−0.007*** (−4.15)	0.010*** (6.86)	0.044*** (12.64)	−0.009*** (−5.86)	0.027*** (10.95)	−0.021*** (−8.83)	0.002 (1.58)
Marriage	0.749*** (40.92)	0.031** (2.31)	0.172*** (7.44)	0.229*** (17.16)	0.203*** (5.43)	0.542*** (11.19)	0.093*** (4.74)
Edu	−0.037*** (−26.42)	−0.048*** (−17.28)	−0.005 (−0.91)	−0.058*** (−29.62)	−0.028*** (−5.00)	−0.080*** (−39.74)	−0.045*** (−23.00)
Work_status	−0.183*** (−11.68)	−0.053*** (−6.94)	−0.050*** (−2.99)	−0.074*** (−11.44)	−0.037*** (−3.10)	−0.186*** (−6.84)	0.011** (2.01)
Familysize	0.051*** (4.15)	0.167*** (27.30)	0.206*** (31.86)	0.159*** (17.39)	0.196*** (31.10)	0.237*** (9.86)	0.159*** (20.12)
Health	−0.397*** (−10.41)	0.009 (1.37)	0.021*** (2.82)	−0.062*** (−7.05)	0.021*** (4.21)	−0.260*** (−14.26)	0.010 (1.01)
Chronic	0.286*** (12.55)	0.011*** (4.45)	0.060*** (7.27)	0.065*** (22.78)	0.068*** (5.88)	0.239*** (12.82)	0.003 (0.66)
Older adult	0.077* (1.90)	−0.067*** (−10.06)	0.004 (0.18)	−0.042*** (−6.22)	−0.091*** (−6.43)	−0.064** (−2.26)	−0.068*** (−4.93)
Pgdp	−0.177* (−1.76)	0.028 (0.95)	0.209*** (4.38)	−0.037 (−0.92)	0.135*** (4.08)	−0.052 (−0.71)	0.017 (0.44)
Constant	6.125*** (5.91)	8.610*** (27.26)	4.744*** (9.37)	9.057*** (22.33)	5.883*** (13.98)	6.229*** (7.99)	7.807*** (21.70)
Observations	69,797	69,797	69,797	69,797	69,797	69,797	69,797
R-squared	0.0339	0.0851	0.0397	0.0815	0.0601	0.0380	0.0699
Number of groups	35,941	35,941	35,941	35,941	35,941	35,941	35,941
Ind	YES	YES	YES	YES	YES	YES	YES
Year	YES	YES	YES	YES	YES	YES	YES

***, **, and * indicate significance at the 1%, 5%, and 10% levels, respectively, with standard deviation in parentheses.

#### 5.2.2 Robustness tests

This paper conducts the following robustness tests for consumption scale.

**Figure 2:**
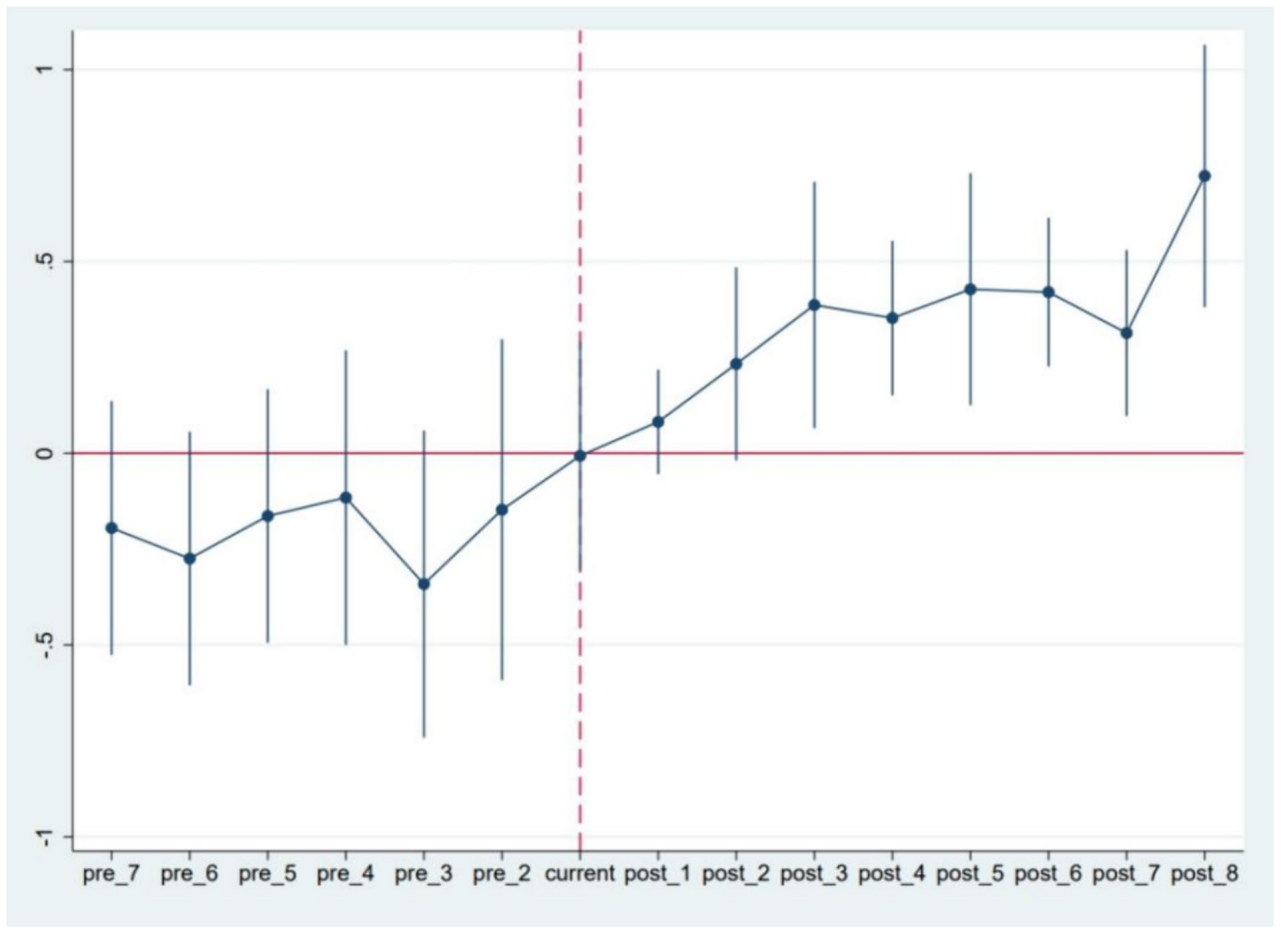
Results of trend analysis of rural residents’ consumption balance.

The first step refers to the parallel trend test. It is crucial to ensure that there are no significant differences in the consumption trends between the treatment group and the control group prior to the implementation of the medical insurance policy. To facilitate this analysis, an event study method was employed to create dummy variables for both the treatment group and time phases occurring before and after the policy’s implementation. The period labeled as pre1 serves as the baseline for regression analysis. The results displayed in Figure 2 indicate that the dummy variable representing the pre-policy period is not statistically significant. This finding confirms that the changes in the dependent variable for both the treatment and control groups adhere to the parallel trend assumption prior to the policy’s introduction. Furthermore, the dummy variables representing post-policy periods (post3 to post8) exhibit positive correlations with the dependent variable at a significance level of at least 5%, suggesting a significant policy effect accompanied by a certain lag.

The second step involves the elimination of anomalous samples.

Given that the sample period selected for this study includes the year 2020 and subsequent years—during which the COVID-19 pandemic had a profound global impact—this public health emergency represents a strictly exogenous external shock affecting both macroeconomic and microeconomic operations. The frequency of medical insurance claims surged significantly following this event, potentially skewing the analysis. If this factor is not controlled for, it may introduce endogeneity issues related to omitted variables. Therefore, columns (1) and (2) of Table 7 present regression analyses conducted after excluding the anomalous samples from 2020 and beyond. Regardless of whether control variables are included, the significance of the core explanatory variable, *integration*, remains consistent with previous findings, indicating robustness in the results.

**Table 7:** Robustness test of consumption scale.

	(1)	(2)	(3)	(4)
Variables	Eliminating COVID-19 impact	Eliminating COVID-19 impact	Municipalities were excluded	After winsorization
Integration	0.015*** (3.12)	0.057*** (16.18)	0.031*** (2.91)	0.032*** (3.04)
Income		0.065*** (20.15)	0.058*** (17.81)	0.059*** (18.58)
Age		0.002*** (3.98)	0.000 (0.40)	−0.000 (−0.33)
Gender		0.065 (0.71)	0.002 (1.33)	0.002** (2.00)
Marriage		0.130*** (6.97)	0.130*** (13.14)	0.148*** (14.54)
Edu		0.053*** (28.44)	0.053*** (23.94)	−0.055*** (−27.14)
Work_status		0.087*** (15.40)	0.076*** (13.99)	−0.075*** (−12.17)
Familysize		0.127*** (15.49)	0.145*** (21.52)	0.145*** (22.41)
Health		0.020*** (2.92)	0.033*** (4.31)	−0.036*** (−4.91)
Chronic		0.034*** (16.70)	0.037*** (27.87)	0.042*** (35.81)
Older adult		0.135*** (12.12)	0.053*** (7.20)	−0.051*** (−7.30)
Pgdp		0.23*** (9.10)	0.019 (0.52)	−0.027 (−0.73)
Constant	9.883*** (2533.45)	11.608*** (43.74)	9.487*** (23.70)	9.564*** (23.69)
Observations	53,926	53,926	67,608	69,797
R-squared	0.0295	0.0687	0.0864	0.0867
Number of groups	35,887	35,887	34,755	35,941
Ind	YES	YES	YES	YES
Year	YES	YES	YES	YES

***, **, and * indicate significance at the 1%, 5%, and 10% levels, respectively, with standard deviation in parentheses.

To further enhance the generalizability of the results, this study also excludes individuals residing in the four municipalities directly governed by the Central Government (Beijing, Shanghai, Tianjin, and Chongqing). These municipalities have a relatively small rural population and distinct socio-economic conditions compared to rural populations in other prefecture-level cities. From the regression results in Column (3) of Table 7, it can be seen that the core explanatory *integration* still has a positive correlation with *consumption* at the significance level of 1%, further affirming the robustness of the findings.

In addition, this paper employs winsorization at the 99th percentile to mitigate the influence of extreme values, producing new regression results. It can be seen from Column (4) of Table 7 that the results are consistent with the main results of Column (5) of Table 5, which is relatively robust.

**Figure 3:**
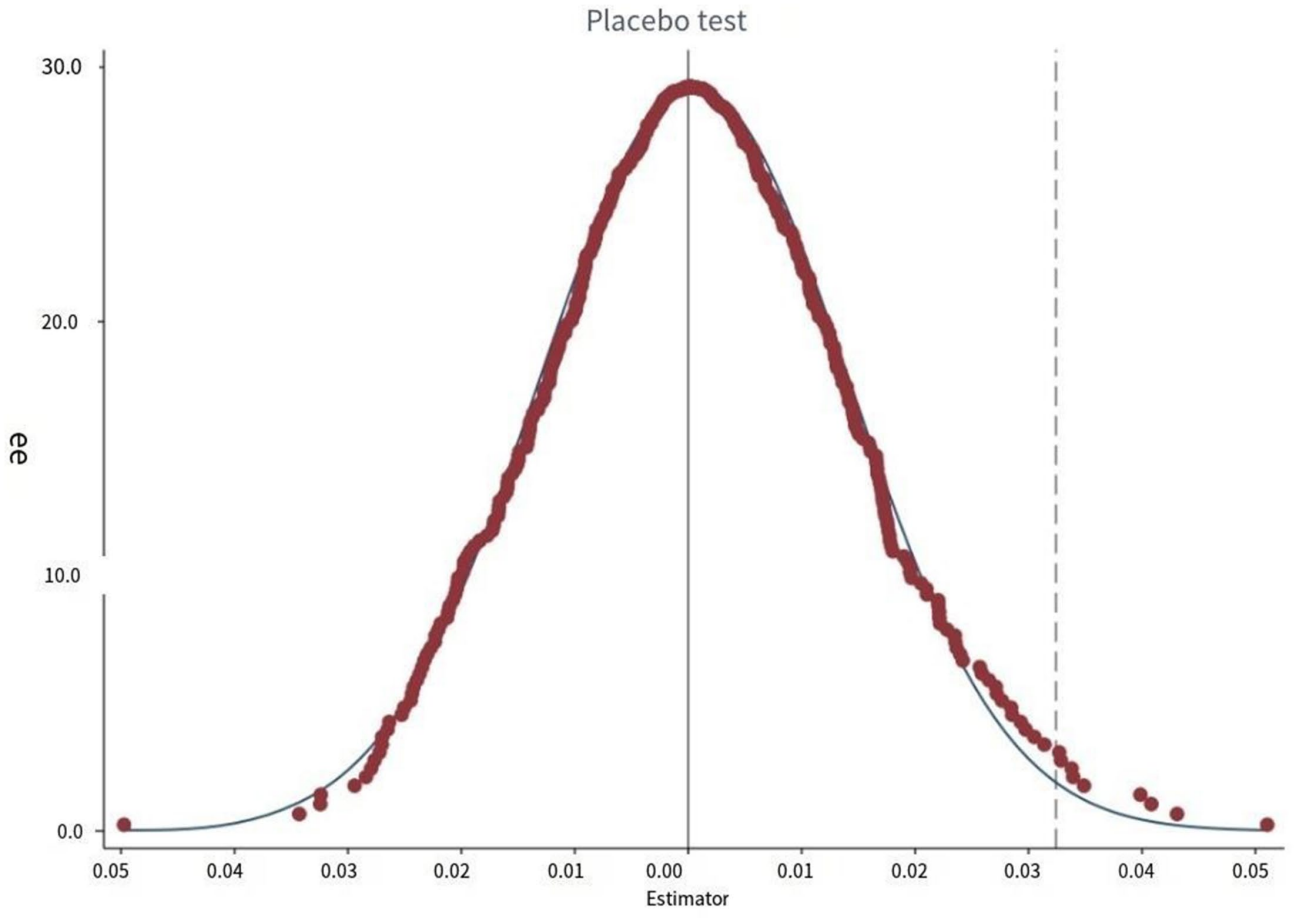
Placebo test of the effect of medical insurance on rural residents’ consumption.

The third robustness check involves a placebo test. To minimize the influence of other potential confounding variables on the relationship between Integration and consumption, this study applies a counterfactual hypothesis method for robustness testing. This involves randomly shuffling the treatment and control groups and selecting an equal number of groups to form a new “treatment group,” which is then subjected to the same policy treatment at the designated time point. A new integration variable is formed by the interaction between the false treatment group and the time dummy variable. After repeating the above experiment 500 times, the coefficient kernel density map is drawn. The results depicted in Figure 3 reveal that the coefficient distribution approximates a normal distribution, indicating that the sample selection is random and free from endogeneity issues due to human interference. Moreover, the actual regression coefficient falls within the small probability rejection domain, confirming that the robustness test is passed.

#### 5.2.3 Heterogeneity analysis

To explore the differential impacts of the URRBMI policy on consumption among middle-aged and older adult rural residents based on gender, marital status, and regional differences, this study employs a group regression approach. Table 8 shows the heterogeneity results of the consumption scale of URRBMI for different samples. In columns (1) and (2), it is evident that the variable Integration lacks significance within the male sample. This may be attributed to the pronounced division of labor between genders in middle-aged and older adult rural households. Generally, the male in the middle-aged and older adult rural family mainly engage in agricultural production or work outside the home to obtain family income, whereas the female take more responsibilities such as taking care of the family. Consequently, a substantial portion of consumption behaviors is driven by the domestic needs of the family. Following the implementation of the URRBMI policy, the impact on consumption is more pronounced among females, as their consumption patterns are directly influenced by the policy.

**Table 8:** Heterogeneity analysis of consumption scale.

	By Gender		By marital status		By Region		
Variables	(1)	(2)	(3)	(4)	(5)	(6)	(7)
	Male	Women	Married	Unmarried	East	Central	West
Integration	0.002 (0.25)	0.090*** (27.20)	0.003 (0.40)	0.192*** (3.47)	0.003 (0.18)	0.030 (1.38)	0.343*** (48.93)
Income	0.050*** (25.47)	0.073*** (17.13)	0.039*** (18.90)	0.185*** (13.18)	0.053*** (15.49)	0.054*** (17.13)	0.076*** (16.80)
Age	−0.029*** (−4.54)	0.013*** (12.93)	0.005*** (6.99)	−0.006 (−0.64)	0.008*** (2.79)	−0.013*** (−9.51)	0.003 (0.99)
Gender			−0.003 (−1.04)	−0.001 (−0.06)	0.017*** (7.24)	−0.007*** (−5.08)	−0.006** (−2.24)
Marriage	−0.046*** (−2.82)	0.210*** (8.78)			0.242*** (10.06)	0.125*** (4.12)	0.065*** (3.12)
Edu	−0.055*** (−45.27)	−0.052*** (−15.35)	−0.024*** (−32.27)	−0.148*** (−20.04)	−0.037*** (−11.90)	−0.068*** (−41.25)	−0.063*** (−23.89)
Work_status	−0.033*** (−6.04)	−0.122*** (−13.55)	−0.052*** (−9.74)	−0.163*** (−9.17)	−0.114*** (−14.98)	−0.043*** (−6.06)	−0.072*** (−4.52)
Familysize	0.135*** (33.98)	0.131*** (12.03)	0.114*** (16.26)	0.167*** (12.43)	0.133*** (20.10)	0.152*** (21.41)	0.151*** (21.62)
Health	−0.041*** (−4.96)	0.013** (2.32)	−0.005* (−1.90)	−0.151*** (−3.86)	0.016 (1.22)	−0.020 (−1.31)	−0.109*** (−14.34)
Chronic	0.052*** (7.47)	0.025*** (9.47)	0.029*** (9.84)	0.089*** (12.54)	0.064*** (5.31)	0.035*** (2.92)	0.025*** (5.10)
Older adult	−0.087*** (−10.55)	−0.157*** (−6.73)	−0.115*** (−10.77)	0.426*** (2.75)	−0.035*** (−2.68)	−0.039 (−1.57)	−0.086*** (−2.89)
Pgdp	−0.001 (−0.03)	−0.227*** (−5.58)	−0.009 (−0.32)	−0.181 (−1.38)	0.091*** (2.91)	−0.122*** (−3.55)	0.015 (0.26)
Constant	11.373*** (19.09)	10.439*** (19.30)	9.452*** (29.91)	10.097*** (7.21)	7.751*** (24.27)	11.459*** (30.81)	8.571*** (10.65)
Observations	33,179	28,192	60,253	9,544	26,584	22,999	20,214
R-squared	0.0821	0.0734	0.0720	0.177	0.0852	0.0871	0.0947
Number of groups	17,331	18,682	31,538	5,429	13,775	11,841	10,325
Ind	YES	YES	YES	YES	YES	YES	YES
Year	YES	YES	YES	YES	YES	YES	YES

***, **, and * indicate significance at the 1%, 5%, and 10% levels, respectively, with standard deviation in parentheses.

In terms of marital status, URRBMI policy has a significant positive correlation with the consumption of unmarried residents. The unmarried individuals in these people often include widowed or never-married individuals of advanced age, resulting in smaller household sizes compared to their married counterparts. The implementation of medical insurance policies can reduce these groups’ expectations of future risks, allowing them to allocate some income for consumption. When this income is distributed among married families, the average change in consumption is negligible; hence, the results in column (3) are relatively smaller.

Compared to the central and eastern regions of China, the URRBMI policy has a significantly positive impact on the consumption of middle-aged and older adult rural residents in the western region. This finding highlights that the enhanced benefits provided by the medical insurance policy effectively reduce the future risk expectations of middle-aged and older adult individuals in the underdeveloped areas of western China. As a result, more income becomes available for consumption, leading to an increased consumption propensity. In contrast, residents in the central and eastern regions—particularly those in the more developed eastern areas—tend to have higher incomes and a more robust local medical security system. Some individuals even opt for supplementary commercial medical insurance, which diminishes the relative impact of basic medical insurance on their consumption patterns.

Further analysis focuses on the consumption quality of middle-aged and older adult women, the unmarried people in rural areas, and the middle-aged and older adult population in underdeveloped regions of western China. Table 9 shows the heterogeneity results of the consumption structure of URRBMI for different samples. The data indicate that the consumption structure among middle-aged and older adult women in rural areas has been optimized. In particular, the growth rate of medical consumption surpasses that of non-medical consumption, while the increase in non-food consumption outpaces that of food consumption. Additionally, the growth in developmental consumption is greater than that of hedonic and subsistence consumption. These trends align with the definition of consumption upgrading previously discussed. The resident medical insurance policy has expanded the scope of medical insurance, increased the medical insurance catalog and treatment guarantee, and many drugs that could not be reimbursed in the past have been included in the insurance coverage. Women who pay more attention to family health will increase the frequency of medical treatment (29). This shift results in a rise in medical consumption that exceeds the growth in non-medical consumption, reflecting a broader focus on health as a marker of improved consumption quality. The significant growth in non-food consumption relative to food consumption, along with the greater increase in development-oriented and enjoyment-oriented consumption compared to subsistence consumption, indicates that middle-aged and older adult women in rural areas are moving beyond basic needs and are currently pursuing higher-level demands, signifying a marked improvement in consumption quality.

**Table 9:** Heterogeneity analysis of consumption structure.

	(1)	(2)	(3)	(4)	(5)	(6)	(7)
Variables	Medical	Non-medical	Food	Non-food	Live	Develop	Enjoy
Female	0.122*** (21.59)	0.070*** (11.01)	0.004 (0.38)	0.104*** (76.43)	0.015 (1.55)	0.182*** (36.59)	0.076*** (13.57)
Unmarried	0.301*** (6.35)	0.183*** (3.07)	0.053 (0.42)	0.285*** (8.04)	0.023 (0.34)	0.064 (0.91)	0.248*** (9.63)
The West	0.035 (0.34)	0.322*** (37.54)	0.145*** (11.42)	0.372*** (32.20)	0.270*** (30.49)	0.224*** (3.36)	0.391*** (68.30)
Other controls	YES	YES	YES	YES	YES	YES	YES
Ind	YES	YES	YES	YES	YES	YES	YES
Year	YES	YES	YES	YES	YES	YES	YES

***, **, and * indicate significance at the 1%, 5%, and 10% levels, respectively, with standard deviation in parentheses.

The unmarried group within the rural middle-aged and older adult population also demonstrates a significant positive effect on non-medical consumption. In these people, medical consumption has decreased markedly, while non-food and hedonic consumption has increased significantly. The unbalanced gender ratio in rural areas, where a majority of widowed or older unmarried individuals are male, contributes to smaller family sizes. Unlike their female counterparts, these men tend to place less emphasis on health. Following the implementation of the URRBMI policy, their future health risk expectations diminish, allowing them to reallocate precautionary savings toward hedonic consumption. With basic medical security provided by the insurance, this group can utilize their income to fulfill various needs, leading to an increase in non-food consumption. Coupled with the increase in hedonic consumption, the quality of consumption of this group has been improved.

For middle-aged and older adult individuals in the Western region, non-medical consumption has seen significant growth, with non-food consumption increasing at a rate greater than that of food consumption. Additionally, hedonic consumption has risen markedly compared to the other categories. The implementation of the URRBMI policy has alleviated uncertainties faced by these people, providing essential medical security. Consequently, these individuals feel more secure in allocating a portion of their income toward higher-order needs, pursuing both hedonic and developmental consumption, thereby improving the structure and quality of their overall consumption.

### 5.3 Results of empirical analysis

The previous section explains in detail the impact of medical insurance for urban and rural residents on the consumption scale of middle-aged and older adult rural residents and further analyzes the impact on the consumption quality. Theoretical analysis has been carried out in the previous mechanism analysis section, which needs to be further tested by an empirical model.

The following are the results of the measurement model, which still takes integration as the core explanatory variable and the control variable remains unchanged, except that the explained variable is changed into the expected household income (Table 10).

**Table 10:** Empirical analysis.

Variables	Exp_income
Integration	0.601*** (0.044)
Ind	YES
year	YES

***, **, and * indicate significance at the 1%, 5%, and 10% levels, respectively, with standard deviation in parentheses.

From the empirical results, this approach is significantly valid, which is consistent with the previous hypothesis 1. The resident medical insurance policy improves the level of social medical security, reduces the future uncertainty of the insured people, and reduces risk expectations, thus releasing part of their precautionary savings originally prepared for medical needs and promoting the expansion of consumption scale.

### 5.4 Discussion of the endogeneity problem

The pilot cities of URRBMI policy were not selected randomly, but the selection was affected by factors such as geographical location, economic development and network development level, which will lead to endogeneity problems, such as missing variables, measurement error, or model misspecification. According to Heckman et al. (30), the propensity score matching-differences-in-differences (PSM-DID) model is introduced in this paper for regression analysis. The results in the table below show that URRBMI can significantly promote consumption. The coefficients and significance of the *consumption* increase slightly, but the signs of the main coefficients remain highly consistent with the time-varying DID regression results (Table 11).

**Table 11:** Impact on consumption scale.

	(1)	(5)	(a)	(b)
Variables	Time-varying DID		PSM-DID	
	Consumption	Consumption	Consumption	Consumption
Integration	0.009 (1.20)	0.032 *** (3.04)	0.076*** (3.66)	0.079*** (3.52)
Income		0.059 *** (18.58)		0.002 (0.71)
Age		0.000 (0.33)		−0.209*** (−9.33)
Gender		0.002** (2.00)		−0.038*** (−2.65)
Marriage		0.148*** (14.54)		0.479*** (3.68)
Edu		0.055*** (27.14)		−0.076*** (−8.67)
Work_status		0.075*** (12.17)		0.076*** (4.18)
Familysize		0.145*** (22.41)		0.189*** (20.61)
Health		0.036*** (4.91)		0.047*** (2.63)
Chronic		0.042*** (35.81)		0.188*** (9.09)
Older adult		0.051*** (7.30)		0.098 (0.92)
Pgdp		0.027 (0.73)		−0.011 (−0.14)
Constant	10.075*** (1123.83)	9.564*** (23.69)	10.883*** (135.41)	23.417*** (20.35)
Observations	69,797	69,797	1,772	1,772
R-squared	0.0448	0.0867	0.0794	0.148
Number of groups	35,941	35,941	1,381	1,381
Ind	YES	YES	YES	YES
Year	YES	YES	YES	YES

***, **, and * indicate significance at the 1%, 5%, and 10% levels, respectively, with standard deviation in parentheses.

Moreover, the quasi-natural experiment is used in this paper and on the basis of controlling fixed effects such as time and region, we use time-varying DID to alleviate the bias of omitted variables and measurement errors on the estimation results. In addition to trying to control the control variables that may cause errors in the estimation results, this paper sets a series of control variables, including the individual and the social levels, and controls the year and individual fixed effects. The probability of endogeneity problems caused by omitted variables and measurement errors is low.

## 6 Conclusion and suggestions

### 6.1 Conclusion

This paper uses the time-varying DID method to discuss the whole-time course of the implementation of URRBMI. The results show that the implementation of the resident medical insurance policy has a significant effect on the consumption scale and structure of the middle-aged and older adult rural residents. In particular, there has been a marked improvement in non-food consumption, developmental consumption, and hedonic consumption, whereas the proportion of food and subsistence consumption relative to total consumption has been significantly curtailed. This shift reflects an overall enhancement in consumption quality. Among the people examined, women, who often bear greater family care responsibilities, are particularly affected by the URRBMI policy, especially regarding non-food, developmental, and hedonic consumption. Medical consumption is impacted more than non-medical consumption, contributing to a larger overall consumption scale and a notable improvement in consumption quality. Furthermore, the consumption patterns of unmarried residents demonstrate a more pronounced response to the medical insurance policy compared to their married counterparts, with significant increases in subsistence, hedonic, and non-food consumption—aligning well with the concept of consumption upgrading. In regional terms, the positive effects of the URRBMI policy on consumption are most pronounced among residents in the western region. Except for medical consumption, all types of consumption have increased, with non-food and hedonic consumption experiencing the most significant growth, thereby satisfying the conditions for consumption upgrading. Overall, the URRBMI policy significantly enhances the consumption scale and structure of vulnerable groups within the rural middle-aged and older adult population, contributing to a reduction in income disparity both within and between rural areas. In addition, this paper also verifies the robustness of the benchmark results through the balanced trend experience, the elimination of abnormal samples, and the placebo test. Empirical analysis and endogeneity problem are taken into account, indicating that the policy can effectively elevate living standards and play a role in poverty alleviation.

In conclusion, URRBMI policy serves to mitigate consumption and welfare inequalities among middle-aged and older adult groups in rural areas and between different regions, enhancing the consumption quality of relatively vulnerable populations. This, in turn, improves living standards and reduces the wealth gap in rural settings.

### 6.2 Policy suggestions and prospects

As a crucial driver of economic growth, consumption plays a vital role in elevating residents’ living standards and enhancing their overall wellbeing. The findings of this study underscore the significant influence of the URRBMI on the consumption scale and quality of middle-aged and older adult individuals in rural areas. Based on the findings, this study proposes policy recommendations from the following four perspectives:

1. Refinement and Expansion of Medical Insurance Coverage for Residents: The study finds that the implementation of URRBMI policy has significantly improved the consumption scale of middle-aged and older adult rural residents, especially for women, unmarried people and residents in the western region and other relatively vulnerable groups. Therefore, the government should focus on strengthening the basic medical insurance system, ensuring fair access to treatment for these vulnerable groups, further alleviating their medical burdens. At the same time, some rural residents give up insurance because of high insurance cost, high threshold of use, insufficient compensation intensity, complex medical procedures in different places, and other reasons in recent years. It is essential to align the medical insurance reform with the rural revitalization strategy, expanding preferential policies for vulnerable populations while ensuring policy sustainability. This will help unlock their consumption potential. The reform of medical insurance should also pay attention to the reasonable optimization of insurance costs, further give full play to the advantages of overall medical insurance, improve the strength of claims settlement, and enhance the sense of gain of the people. Local governments should actively publicize the interpretation of the basic medical insurance policy to improve the understanding of rural residents to the policy, improve the settlement of medical treatment in other places, and promote the efficiency of settlement.
2. Synergy Between Health Insurance and Poverty Alleviation Policies: The results show that the medical insurance policy not only improves the medical consumption of rural residents but also promotes the growth of non-medical consumption, reflecting the overall effect of the policy. Therefore, the government should foster coordination between residents’ medical insurance and other poverty alleviation initiatives, such as social assistance and education subsidies. By creating a synergistic relationship among these policies, the comprehensive effects of poverty alleviation can be maximized, effectively increasing the income and consumption scale of rural residents living in poverty.
3. Policy Optimization and Consumption Upgrading: The results show that the medical insurance policy has promoted the proportion of non-subsistence consumption of rural residents, and the quality of consumption has been improved. In order to consolidate and expand this result, the government should innovate relevant policy mechanisms, intensify support for the supply of goods and the construction of consumer facilities in rural areas. Additionally, by optimizing the rural business environment, improving financial services in rural areas, encouraging preferential treatment for rural areas on e-commerce platforms, and improving logistics channels, the government can provide platforms and opportunities for the upgrade of resident consumption. This will stimulate the consumption willingness of rural residents, especially the older adult, and promote the continuous improvement of the quality of rural consumption.
4. Emphasizing Policy Coordination to Narrow Development Gaps Among Different Groups: The research shows that the resident medical insurance policy makes the consumption growth and quality improvement of middle-aged and older adult rural residents different by gender, marital status, and region. Therefore, the government should focus on the coordination of various policies, strengthen targeted support for specific groups, and create a synergistic effect among policies that benefit the public, such as medical insurance, educational funding, and entrepreneurship and employment support. This effort aims to continuously reduce the gaps in income levels and quality of life between different populations and regions. Specifically, for the female population, it is recommended to include special support for women in the medical insurance policy, such as expanding health protection and family medical services for women. For unmarried residents, more medical and social welfare support should be considered to compensate for their lack of family support. For residents in the western regions, it is suggested to increase medical insurance funding and policy support to maintain and expand the positive impact, with a particular focus on the growth of non-food consumption and consumption for enjoyment. For the impoverished population, it is essential to further implement targeted poverty alleviation measures, integrate the medical insurance policy with other poverty alleviation projects, enhance the consumption capacity and living standards of the poor, and promote broader poverty reduction effects.

In summary, to fully leverage the comprehensive benefits of the basic medical insurance system for residents, it is essential to continuously improve the policy framework, promote a comprehensive improvement in income and living standards among the rural poor, and contribute to the realization of rural revitalization and the goal of common prosperity. As evidenced above, the urban and rural resident medical insurance policy can effectively alleviate consumption inequalities within rural areas and between regions, providing a theoretical basis for policy formulation.

In addition, the ongoing medical insurance reform is making significant strides. The current planning of basic medical insurance has initially realized the unification of URRBMI and the NCMS. Moving forward, there are two primary directions for enhancing the urban–rural integrated medical insurance system. The first direction involves expanding the coverage of the integrated medical insurance scheme for both urban and rural residents, alongside promoting provincial pooling. Chinese government encourage regions where conditions permit to implement province-level pooling of basic medical insurance. Provincial-level pooling requires a relatively high degree of balanced development of the economy, healthcare, and medical insurance systems within a province. At present, only four municipalities directly under the Central Government and some underdeveloped provinces in western China have basically reached the level of provincial-level pooling of urban and rural medical insurance. The second direction focuses on further integrating URRBMI with Urban Employee Medical Insurance (UEMI), aiming to establish a comprehensive medical insurance system that serves the entire population. For instance, Guangdong Province began integrating serious illness insurance for both employees and urban–rural residents in 2016. This initiative is grounded in the province’s economic capacity, as it has achieved the standards of a moderately developed city, coupled with a progressively narrowing policy gap. The urban–rural integrated medical insurance system can further deepen the reform of the medical security system, promote the integration of urban and rural social security, and promote the convergence and balance of standards among urban and rural areas, regions, industries, and populations. In the future, the impact of medical insurance policy reform on consumption can also be further discussed from these two aspects.

## Data Availability

Publicly available datasets were analyzed in this study. This data can be found at: https://charls.charlsdata.com/ and https://www.pkulaw.com/.

## Footnotes

^1^The term has been repeatedly mentioned in China, which refers to the dynamic system of smooth circulation in which production, circulation, distribution and consumption are organically connected with meeting the needs of domestic people for a better life, supported by the domestic division of labor system and industrial system, supplemented by the international circulation.

^2^Data from the National Bureau of Medical Insurance.

^3^ https://www.pkulaw.com/

^4^CHARLS is a large-scale microscopic database on health, work and pension issues of middle-aged and older adult people over 45 years old in China, which is led by the National School of Development of Peking University. It contains abundant information on health, income and expenditure, social security and other issues at the individual and family levels. CHARLS contains specific questions on household consumption expenditure, which provides data support for this study. The CHARLS survey was conducted in 28 provinces, 150 municipalities or regions, and 450 villages or urban communities in China from 2011 to 2012 using stratified random sampling method. The second wave of the survey was conducted in 2013, the third wave in 2015, and the fourth wave in July 2018，the fifth wave in 2020. This paper focuses on rural residents, that is, individuals holding rural hukou at the time of the survey.

^5^Sources: statistical yearbooks and bulletins of various provinces and cities.

## References

[1] Chamon M Prasad E. Why are saving rates ofurban households in China rising? American Econ J Macroeco. (2010) 2:93–130. doi: 10.1257/mac.2.1.93

[2] Ge S Yang DT Zhang J. Population policies, demographic structural changes, and the Chinese household saving puzzle. Eur Econ Rev. (2018) 101:181–209. doi: 10.1016/j.euroecorev.2017.09.008

[3] He H Huang F Liu Z Zhu D. Breaking the “iron rice bowl:” evidence of precautionary savings from the Chinese state-owned enterprises reform. J Monet Econ. (2018) 94:94–113. doi: 10.1016/j.jmoneco.2017.12.002

[4] Vatsa P Li J Luu PQ Botero-R JC. Internet use and consumption diversity: evidence from rural China. Rev Dev Econ. (2023) 27:1287–308. doi: 10.1111/rode.12935, PMID: 39166077

[5] Liu K. Insuring against health shocks: health insurance and household choices. J Health Econ. (2016) 46:16–32. doi: 10.1016/j.jhealeco.2016.01.002, PMID: 26836108

[6] Liu Y Si X. Consumption insurance pattern differences between China and the US: the role of self-and external insurance. Financ Res Lett. (2023) 58:104384. doi: 10.1016/j.frl.2023.104384

[7] Yingqian TANG Rong FU Noguchi H. Impact of medical insurance integration on reducing urban-rural health disparity: evidence from China. Soc Sci Med. (2024) 357:117163. doi: 10.1016/j.socscimed.2024.117163, PMID: 39121565

[8] Darkwah F. Does free health insurance improve health care use and labour market outcomes of the elderly in Ghana? J Econ Ageing. (2022) 23:100418. doi: 10.1016/j.jeoa.2022.100418

[9] Levine D Polimeni R Ramage I. Insuring health or insuring wealth? An experimental evaluation of health insurance in rural Cambodia. J Dev Econ. (2016) 119:1–15. doi: 10.1016/j.jdeveco.2015.10.008, PMID: 39306736

[10] Ren Y Zhou Z Cao D Ma BH Shen C Lai S. Did the integrated urban and rural resident basic medical insurance improve benefit equity in China? Value Health. (2022) 25:1548–58. doi: 10.1016/j.jval.2022.03.007, PMID: 35514010

[11] Wagstaff A. The economic consequences of health shocks: evidence from Vietnam. J Health Econ. (2007) 26:82–100. doi: 10.1016/j.jhealeco.2006.07.001, PMID: 16905205

[12] Dunn A. Health insurance and the demand for medical care: instrumental variable estimates using health insurer claims data. J Health Econ. (2016) 48:74–88. doi: 10.1016/j.jhealeco.2016.03.001, PMID: 27107371

[13] Wang Y Shi J Yao Y Sun W. The impact of health insurance on job location choice: evidence from rural China. J Comp Econ. (2022) 50:569–83. doi: 10.1016/j.jce.2022.01.001

[14] Chen H Ding Y Tang L Wang L. Impact of urban–rural medical insurance integration on consumption: evidence from rural China. Econ Anal Policy. (2022) 76:837–51. doi: 10.1016/j.eap.2022.09.018, PMID: 39267656

[15] del Valle A. The effects of public health insurance in labor markets with informal jobs: evidence from Mexico. J Health Econ. (2021) 77:102454. doi: 10.1016/j.jhealeco.2021.102454, PMID: 33784539

[16] Zhao W. Does health insurance promote people’s consumption? New evidence from China. China Econ Rev. (2019) 53:65–86. doi: 10.1016/j.chieco.2018.08.007

[17] Wagstaff A Lindelow M Jun G Ling X Juncheng Q. Extending health insurance to the rural population: an impact evaluation of China’s new cooperative medical scheme. J Health Econ. (2009) 28:1–19. doi: 10.1016/j.jhealeco.2008.10.007, PMID: 19058865

[18] Wang Z Wu Z Yuan Y. We’ve got you covered! The effect of public health insurance on rural entrepreneurship in China. J Public Econ. (2024) 235:105150. doi: 10.1016/j.jpubeco.2024.105150

[19] Sun JY. Welfare consequences of access to health insurance for rural households: evidence from the new cooperative medical scheme in China. Health Econ. (2020) 29:337–52. doi: 10.1002/hec.3985, PMID: 31814170

[20] Sheu JT Lu JFR. The spillover effect of National Health Insurance on household consumption patterns: evidence from a natural experiment in Taiwan. Soc Sci Med. (2014) 111:41–9. doi: 10.1016/j.socscimed.2014.04.006, PMID: 24747377

[21] Callaway B Sant’Anna PHC. Difference-in-differences with multiple time periods. J Econ. (2021) 225:200–30. doi: 10.1016/j.jeconom.2020.12.001

[22] Goodman-Bacon A. Public insurance and mortality: evidence from Medicaid implementation. J Polit Econ. (2018) 126:216–62. doi: 10.1086/695528, PMID: 38124506

[23] Goodman-Bacon A. Difference-in-differences with variation in treatment timing. J Econ. (2021) 225:254–77. doi: 10.1016/j.jeconom.2021.03.014

[24] Friedman M. (1957) ‘A theory of the consumption function. Princeton University Press.’ Princeton, NJ. 122–124

[25] Maslow AH. A theory of human motivation. Psychol Rev. (1943) 50:370–96. doi: 10.1037/h0054346, PMID: 39304901

[26] Beck T Levine R Levkov A. Big bad banks? The winners and losers from bank deregulation in the United States. J Financ. (2010) 65:1637–67. doi: 10.1111/j.1540-6261.2010.01589.x

[27] Aguiar M Hurst E. Deconstructing life cycle expenditure. J Polit Econ. (2013) 121:437–92. doi: 10.1086/670740

[28] Carroll CD Crawley E Slacalek J Tokuoka K White MN. Sticky expectations and consumption dynamics. Am Econ J Macroecon. (2020) 12:40–76. doi: 10.1257/mac.20180286, PMID: 37877701

[29] Nair SC Al Saraj Y Sreedharan J Vijayan K Ibrahim H. Health literacy levels in patients with type 2 diabetes in an affluent gulf country: a cross-sectional study. BMJ Open. (2023) 13:e069489. doi: 10.1136/bmjopen-2022-069489, PMID: 36746537 PMC9906167

[30] Heckman J Ichimura H Todd P. Matching as an econometric evaluation estimator. Rev Econ Stud. (1998) 65:261–94. doi: 10.1111/1467-937X.00044, PMID: 38625175

[31] Sun J Li C. Research on consumption upgrading path combining demand side and supply side. Journal of Renmin University of China. (2022) 52–62.

[32] Xiaohua W Xiaoke M Qian H. Has the use of digital finance fully released the power of domestic demand in rural consumption? China Rural Economy. (2022) 21–39.

